# Ultrasonic Measurement of Common Carotid Intima-Media Thickness in Type 2 Diabetic and Non-Diabetic Patients

**DOI:** 10.5812/iranjradiol.7564

**Published:** 2012-06-30

**Authors:** Ahmad Alizadeh, Ali Roudbari, Abtin Heidarzadeh, Ali Babaei Jandaghi, Maryam Bani Jamali

**Affiliations:** 1 Department of Radiology, Poursina Hospital, Guilan University of Medical Sciences, Rasht, Iran; 2 Department of Neurology, Poursina Hospital, Guilan University of Medical Sciences, Rasht, Iran; 3 Department of Community Medicine, Guilan University of Medical Sciences, Rasht, Iran; 4 General Practitioner, Guilan University of Medical Sciences, Rasht, Iran

**Keywords:** Arteriosclerosis, Diabetes Mellitus, Carotid Arteries, Ultrasonography

## Abstract

**Background:**

Diabetes mellitus is a widespread disease. Its vascular complications can be characterized by arteriosclerosis formation in carotid arteries. Due to its delayed diagnosis resulting in more complications in Iran, it seems that screening diabetic patients is mandatory.

**Objectives:**

The aim of this study was to compare the intima-media thickness (IMT) of carotid artery in diabetic and non-diabetic patients.

**Patients and Methods:**

This is a cross-sectional study, which included 80 participants (40 diabetics and 40 non-diabetics). By using ultrasound, bilateral IMTs of the distal carotid were measured and the data were analyzed using ANOVA and multivariate regression tests in SPSS 14.

**Results:**

The mean IMT was 0.97 in diabetic patients and 0.63 in non-diabetics (P < 0.001). Age and gender had significant positive effects on the increase of IMT (P < 0.05 and P < 0.005, respectively for age and gender). Past medical history of coronary heart disease (CHD) and cerebrovascular accident (CVA) in diabetes is associated significantly with an increase in IMT (P =0.019 and 0.027 respectively). Other confounding variables such as smoking, history of hypertension (HTN) and hyperlipoproteinemia (HLP) in diabetic patients showed no significant relationship with the increase of IMT.

**Conclusions:**

Although measuring the IMT of the carotid artery by sonography is a useful tool for screening diabetic patients, more studies are needed for determining how to use these measurements in promoting the patients outcomes.

## 1. Background

Diabetes mellitus is a widespread disease. Type 2 diabetes is the common form of diabetes and the cause of 90 % of diabetes in the world (1, 2). It is expected that the number of diabetics will increase to 300 million patients in 2025 (3). Risk factors for type 2 diabetes are genetic factors, gender, age, race, obesity, inactivity, diet, stress, insulin resistance, glucose intolerance and pregnancy factors such as parity and gestational diabetes (3). Diabetes has two vascular complications; one of which are microvascular complications including retinal and glomerular diseases, strongly communicated with the duration and intensity of hyperglycemia (4, 5). In addition, various studies showed that different patients with similar severity of hyperglycemia have different microvascular complications and these observations indicate that genetic differences effect obviously (3). Unlike microvascular complications that occur only in diabetic patients, macrovascular diseases also occur in healthy individuals. In addition, in diabetics, macrovascular complications develop more quickly and the number of involved vessels is higher (6, 7). In diabetic and non-diabetic patients, arteriosclerosis begins with endothelial damage or dysfunction (8). Considering the diagnosis age of diabetes in our country, which is in the fourth decade which is later than Western countries, the prevalence of diabetes complications is higher. Arteriosclerosis particularly in coronary and carotid arteries is one of the most common complications in diabetes. Nowadays, there are different techniques for evaluating carotid artery arteriosclerosis and demonstrating the extent of lesions. One of these methods is ultrasound which is a safe, non-invasive and cost effective method for evaluating the lumen and walls of particular arteries including the aorta, carotid and femoral artery (9, 10). Various studies have shown that ultrasound has the ability to evaluate the intima-media carotid changes (11) and histological assessments have demonstrated that intima and adventitia thickness in ultrasound images may be better diagnosed. In a study performed in Iran, the mean thickness of intima-media without risk factors was mentioned less than the global average (12).

## 2. Objectives

While there is no study available in our country that has investigated diabetes as a risk factor in these cases and with regard to cultural, racial and geographical differences as well as the delay in diagnosis resulting in a higher rate of complications, the necessity of having a mean intima-media thickness (IMT) in diabetic patients in comparison with non-diabetics to perform the screening program is felt. Therefore, in this study, we attempted to compare IMT in carotid arteries in diabetic and non-diabetic patients.

## 3. Patients and Methods

This is a cross-sectional study that included 80 participants (40 diabetics and 40 non-diabetics) referred to the radiology department of Poursina hospital in Rasht/Iran 2007-08. Inclusion criteria for diabetic patients were a fasting blood sugar (FBS) higher than 126 and a blood sugar (BS) higher than 200 with polyuria and polydipsia and having one of the items such as a history of hospital records, clinical tests or using metformin and glibenclamide. Non-diabetics were those who had a normal FBS test and none of the items above. Exclusion criteria were patient’s dissatisfaction, type 1 diabetes or congenital vascular disease. Participants were matched for age and gender. Hawk 2002 gray-scale ultrasound with a special multi-frequency linear probe (V-K medical company, Denmark) was used. We assessed the common carotid arteries 3cm proximal to the bulb in the sagittal cut. The normal thickness of intima-media were considered less than one millimeter. For measuring the thickness of the carotid artery, the patient should lie down in the supine position with the head slightly hyperextended and the radiologist should sit over the patient. In addition, patients should put down their shoulder during ultrasound as much as possible to expose higher levels of the neck and should not move their head. Data were analyzed by ANOVA and multivariate regression tests in SPSS 14.

## 4. Results

The mean age of all 80 participants was 50.97 ± 6.55 years (51.3 years for diabetics and 50.6 years for non-diabetics). The mean IMT is shown in Table 1. In addition, statistical analysis of covariance determined that age is associated with the increase of IMT. The mean IMT was 1.10 in diabetic patients older than 50 years and 0.70 in less than 50-year-olds. Furthermore, the mean IMT in non-diabetic patients older than 50 and lower than 50 years was 0.68 and 0.57, respectively. In our study, the IMT in diabetics and non-diabetics was 0.97 and 0.63, respectively (P < 0.001). Results conducted by statistical covariance found that the history of cerebrovascular accident (CVA) and coronary heart disease (CHD) in diabetic patients significantly associate with the increased IMT (P = 0.027 and P = 0.019, respectively) (Table 2 and Table 3) However, other variables including smoking, history of hypertension (HTN) and hyperlipoproteinemia (HLP) in diabetics did not show a significant association with the increased IMT. Multivariate regression analysis was performed on the variables which is shown in Table 4.

**Table 1:** Basic Characteristics in Diabetic and Non-Diabetic Patients

	** Diabetic **	** Non-Diabetic **
Age , mean ± SD	51.33 ± 8.89	50.6 ± 10.28
Left IMT ^a^ , mean ± SD	0.97 ± 0.45	0.67 ± 0.18
Right IMT, mean ± SD	0.98 ± 0.38	0.66 ± 0.23
Mean IMT, mean ± SD	0.98 ± 0.4	0.63 ± 0.23
Diabetes duration, mean ± SD	10.8 ± 6.66	-
Sex, male/female, %	50/50	50/50
Coronary diseases, %	25	5
CVA ^a^, %	10	2.5
HTN ^a^, %	45	22.5
HLP ^a^, %	45	27.5
Smoking, %	27.5	22.5

^a^Abbreviations: CVA, cerebrovascular accident; HLP, hyperlipoproteinemia; HTN, hypertension; IMT, intima-media thickness

^a^Abbreviations: ANOVA, analysis of variance; CVA, cerebrovascular accident

^a^Abbreviations: ANOVA, analysis of variance; CAD, coronary artery disease

^a^Abbreviations: CVA, cerebrovascular accident; DM, diabetes mellitus; HLP, hyperlipoproteinemia; HTN, hypertension; TDM, time of diabetes mellitus

**Table 2:** Mean and Standard Deviation of Carotid Intima-Media Thickness in Diabetic and Non-Diabetic Patients With and Without CVA

	**CVA ^a^**	**Carotid Intima Media Thickness, Mean ± SD**	**P, ANOVA ^a^**
Diabetics	yes	1.22 ± 0.18	0.27
	no	0.96 ± 0.40	
Non-diabetics	yes	0.80	
	no	0.62 ± 0.16	

**Table 3:** Mean and Standard Deviation of Carotid Intima-Media Thickness in Diabetic and Non-Diabetic Patients with and Without CAD

	**CAD ^a^**	**Mean ± SD**	**P, ANOVA ^a^**
Diabetics	yes	1.13 ± 0. 40	0.019
	no	0.92 ± 0. 38	
Non-diabetics	yes	0.74 ± 0.10	
	no	0.62 ± 0.16	

**Table 4:** Multivariate Regression Analysis on Variables

	**Coefficient**	**Standard Error**	**Z**	**P > Z**	**95% CI**	
DM ^a^	0.344	0.068	5.08	< 0.001	0.209	0.479
TDM ^a^	0.006	0.032	0.18	0.857	-0.058	0.07
Age	0.02	0.025	0.81****	0.418	-0.028	0.068
Gender	-0.112	0.413	-0.27	0.786	-0.922	0.698
Coronary	0.012	0.512	0.02	0.981	-0.991	1.016
CVA ^a^	-0.015	0.642	-0.02	0.982	-1.273	1.244
HTN ^a^	-0.165	0.371	-0.44	0.657	-0.893	0.563
HLP ^a^	0.067	0.382	0.17	0.861	-0.683	0.816
Smoking	0.264	0.440	0.6	0.549	-0.599	1.126
Constant	-0.996	1.333	-0.75	0.455	-3.608	1.615

## 5. Discussion

Coronary artery disease (CAD) and CVA are major causes of morbidity and mortality among diabetic patients. The probability of cardiovascular disease is two-fold in diabetic males and it should be mentioned that vascular diseases are observed more in diabetics. Even after controlling confounding factors such as age, HTN, smoking and HLP, diabetes is still an independent risk factor for cardiovascular disease. In fact, all the risk factors mentioned above contribute less than 25% of the cardiovascular disease incidence in diabetic patients. Increased IMT is a risk factor for CAD which increases the incidence of cardiovascular events. In our study, the relation between CAD and the increase of IMT in diabetic patients showed significant differences. Therefore, ultrasound measurement of carotid IMT is a suitable method for evaluating subclinical arteriosclerosis. Our findings showed that the IMT of carotid is significantly higher in diabetics (0.97 mm) in comparison to non-diabetics (0.63 mm). This finding was similar to Pollex et al. and Temelkova-Kurktschiev et al.’s study (13, 14). In addition, we found that a past medical history of CVA is accompanied with an increased IMT in diabetics. These findings were the same in Salasidis et al. and Salonen et al.’s study (15, 16). In our study, confounding factors such as smoking, HLP and HTN were also considered, but despite their positive role in non-diabetics, findings did not show a significant association with increased IMT. Unlike the findings above, Temelkova-Kurktschiev and colleagues found a significant difference. This difference may have occurred due to the fact that in our study, most patients with HTN and HLP had been treated by drugs and their disease was under control or maybe due to differences in the definition of smoking between studies. Therefore, it is recommended that regardless of the factors mentioned above, diabetics should be screened. Based on findings in this study, factors such as age, gender and duration of diabetes have a significant association with the increase of IMT which was similar to Lee et al.’s study (17). This issue mentions that screening should be considered in male and old individuals and those with more than 10 years diabetes diagnosis.

Based on the results and in spite of the association between CVA and CAD with the increase of IMT in diabetics, measurement of IMT by sonography is a useful tool for screening diabetic patients. However, there is still no investigation showing how measurement has an effect on the patients recovery and more studies are necessary for determining how to use these measurements in promoting patient outcome.
